# Task allocation in a cooperative breeder reflects current needs, not early-life experience

**DOI:** 10.1038/s41598-025-20618-1

**Published:** 2025-10-22

**Authors:** Océane Vanessa Ferreira, Barbara Taborsky

**Affiliations:** Division of Behavioural Ecology, Institute of Ecology and Evolution, University of Bern, Bern, Switzerland

**Keywords:** Cooperative breeding, Division of labour, Early life, Flexibility, *Neolamprologus pulcher*, Task specialisation, Developmental biology, Ecology, Evolution, Zoology

## Abstract

Division of labour is often perceived as one of the major evolutionary transitions in social groups. Specialisation of individuals in different tasks performed in a territory can increase group efficiency in cooperative breeders and eusocial insects. Nevertheless, environmental unpredictability should select for the presence of generalist individuals, which can flexibly perform all tasks in the territory. Whether individuals should develop as specialists or generalists has been intensively studied to understand ecological influences on the evolution of complex societies. Experiences occurring during early life are crucial for the acquisition of appropriate behaviours in a social context. We examined later-life task performance of helpers of the cooperatively breeding cichlid *Neolamprologus pulcher*, which had received different early life experiences of helping needs. We demonstrated that neither repeated early life experience with the need to defend against a predator, nor the need for territory maintenance influenced current task performance. Instead, the current needs for help, such as for egg care, predator defence, and territory maintenance influenced task performance of helpers. Therefore, our results suggest that prioritising present requirements over early life experiences shapes task distribution among cooperative breeders.

**Supplementary Information:**

The online version contains supplementary material available at 10.1038/s41598-025-20618-1.

## Introduction

 Sociality and cooperation within social groups are prevalent throughout the animal kingdom, bearing significant implications for the success of defence against predators^1^ and reproductive success^2^. A defining feature of many highly social, cooperative systems is division of labour, where different individuals perform different tasks in a group, either spontaneously or based on temporal or permanent task specialisation^3–6^. If task specialisation occurs, it enables individuals to focus on particular tasks, thereby reducing the individual costs associated with task switching and enhancing group efficiency^3,6^. Moreover, if individuals of a social unit depend on the tasks performed by others, higher forms of social complexity such as eusociality can evolve^3^.

Task specialisation has been described in a range of animal social organisations, such as cooperative hunters^7^, collective nest-building species^8^ and most prominently in eusocial insects^5,6,9,10^. In cooperatively breeding species, individuals forego their own reproduction to assist dominant members in raising the latters’ offspring^11^. Division of labour between dominants and subordinates has been evidenced in various species^11–15^. In contrast, evidence for task specialisation among helpers of cooperative breeders is limited. Several studies report a lack of longer-lasting individual specialisation—unlike the permanent castes observed in eusocial insects^4^. Instead, helping behaviours often vary with age or body size^16,17^, consistent with age polyethism, a flexible form of task allocation based on age or developmental stage^18^. Specialist and generalist individuals can also coexist within groups^19,20^, which most likely enhances flexibility in task performance at the group level. Specifically, generalists can enhance group survival in unpredictable environments and small groups through their ability to perform all tasks, albeit with some loss of efficiency^21,22^.

Generalist workers in eusocial insects are often characterised as the flexible source of a colony, as they are capable of adjusting their tasks as needed^22,23^. Nevertheless, specialised workers can also express flexibility by adjusting their contributions to the colony’s requirements. For instance, foragers increase their workload in response to the colony’s nutritional status^24^, or to variations in brood number and colony size^25^. Additionally, plasticity of helping phenotypes is well-documented in eusocial insects. Workforce availability can influence worker development in ants, leading to precocious foraging, or even the reversal of roles from foraging back to nursing^26–28^. Similarly in honeybees, nutritional stress results in precocious foraging^29^, and removal of young workers leads to behavioural reversion^30^. Consequently, the mechanisms underlying flexible adjustments in task contributions have been extensively investigated, including physiological thresholds and spatial-encounter mechanisms^21,31,32^.

In cooperative breeders, flexibility in helping behaviours has also been extensively documented. Helpers often increase their workload when group size decreases, or when other group members are unable to help^19,33,34^. Likewise, helpers flexibly adjust their contribution based on predation pressure^35^ and the presence of offspring or eggs^36,37^. Although cooperative breeders lack a distinct differentiation between specialists and generalists, individual characteristics can explain differences in helping effort and task allocation. These differences are likely to contribute to division of labour, thereby enhancing group flexibility. Key individual traits influencing helping behaviours include age^13,16,17^, size and body mass^17,38,39^, and sex^12,20^. Nonetheless, in environments where reliable cues are available, individuals may benefit from integrating information during their early life, leading to the development of a specialised helping phenotype adapted to the prevailing environmental conditions^6,40^. Yet, we still know surprisingly little about the mechanisms generating and maintaining task specialisation or generalism in social species^41^.

Experiences occurring from the prenatal period to sexual maturity are crucial for the acquisition of appropriate behaviours, with short-term and long-term consequences on reproductive success and survival^42^. In eusocial insects, brood nest temperature during the pupal stage influences task specialisation and working contribution later in life^43,44^. In cooperative breeders, the early social environment significantly influences the expression of social behaviours^45–47^ and caste differentiation^48–50^. In addition, early-life factors such as group size, predation exposure, and competition have been shown to influence reproductive strategies and helping phenotypes^51–53^. Hence, early life plays a crucial role in shaping a broad range of behaviours in cooperative breeders, including helping behaviours.

In the cooperatively breeding cichlid *N. pulcher*, individuals form stable groups defending piles of rocks and stones or shell beds as their territory, where they use the clefts between the structures as shelters^54^. Groups include a dominant pair that produces clutches in a central breeding shelter of the territory. These groups are supported by 1 to 20 brood care helpers, which may be offspring of the dominants or unrelated individuals that have dispersed from neighbouring territories^55^. Helpers perform a range of tasks within the territory, including cleaning the eggs, maintaining the territory [such as clearing shelters of sand, snails and debris (“digging” behaviour)], and defending the group territory against heterospecifics and unfamiliar conspecifics^38,56^.

In *N. pulcher*, the presence of older group members and predators prenatally or during early life induces developmental plasticity of a range of social behaviours^47,52,57^, including helping propensity^52,58^. Moreover, in *N. pulcher*, task performance has been shown to depend on size, the location where a task is encountered, and dominance status^35,38,59^. Thus, the results of previous studies suggest that task distribution among helpers (i.e. division of labour) may be determined by context-dependent factors^6^, and varies strongly between social groups (Ramesh & Taborsky, *in prep*). Because of the rich information available on the developmental plasticity of behaviours and on factors influencing task performance in this species, *N. pulcher* constitutes an appropriate model to examine whether early-life experiences can give rise to task specialisation.

Here, we investigated whether early-life experiences influence the development of specialist and generalist helpers in *N. pulcher*. To this end, we experimentally reared juveniles for a period of five months by repeatedly exposing them to different helping demands in their early social environment, but without directly assessing their helping behaviour during this phase. Helpers were reared under one of three experimental conditions simulating repeated helping demands for: (1) territory maintenance, in particular digging away sand (referred to as the ‘specialists in digging’ treatment), (2) egg-predator defence (‘specialists in egg-predator defence’ treatment), or (3) both tasks (‘generalists’ treatment). Each treatment was replicated across five family groups (i.e. 15 family groups in total), and a total of 30 helpers (10 per rearing treatment) were used in this study. Once adults, individuals were introduced to a new social group containing a dominant pair and exposed to four test situations mimicking simultaneous demands of varying intensity for territory maintenance and defence. We assessed their helping phenotype by examining their degree of specialisation and task preference. In addition, we tested their flexibility in helping contributions by evaluating how they adjusted their behaviour in response to changing task demands. We expected that, in addition to helping behaviour per se^52,58^, also the specialisation for digging or defence or for becoming a generalist performing both tasks likewise is shaped by early-life experience in *N. pulcher*. Consequently, we predicted that fish, which experienced multiple cooperative tasks during early life, would adjust their helping contributions flexibly across both tasks. In contrast, fish exposed to only one task during early life were expected to flexibly adjust their contributions primarily to different demand intensities within that task.

## Results

### Helping phenotype

All individuals defended against the egg-predator at least once. Only one individual (from the ‘generalists’ treatment) never dug.

**Fig. 1:**
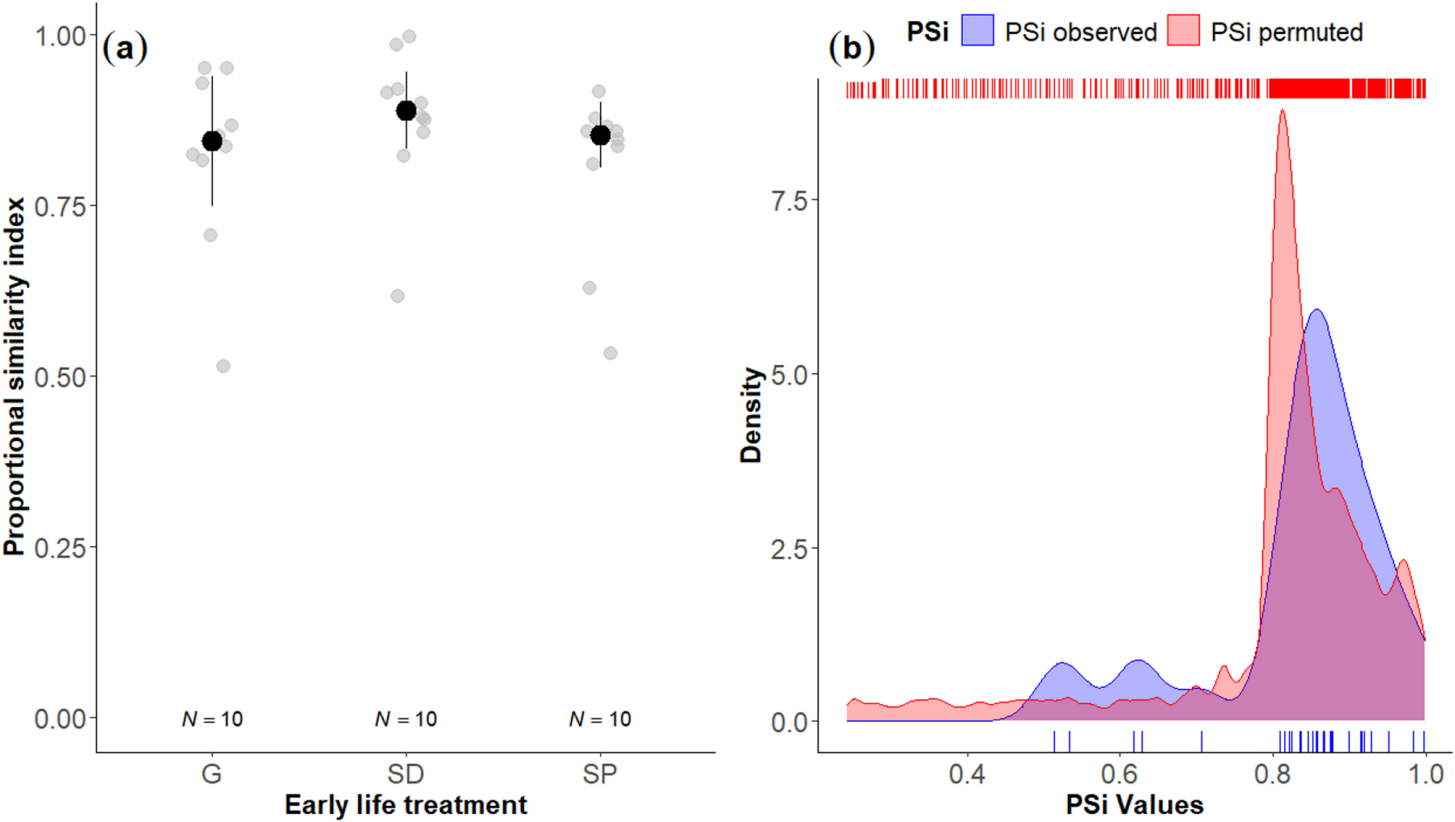
Proportional similarity index (PSi) results. (a) PSi according to the three early life treatments (G: generalist; SD: specialist in digging; SP: specialist in egg-predator defence). Grey dots represent individual data points. Total sample sizes are given below the dots. (b) Density and rug plots of observed and permuted PSi values of the entire study population. Rug plots at the bottom (blue) and top (red) of the figure indicate the individual data points for the observed and permuted PSi values, respectively.

The influence of the early life treatment on the helping phenotype (specialist vs. generalist) was assessed by comparing the proportional similarity index (PSi) across treatments. The early life environment did not significantly influence the PSi (PERMANOVA: *F* = 0.721; *df* = 2, 12; *P* = 0.492; 10,000 permutations, Fig. 1(a)), indicating that the early life did not affect the degree of specialisation of the focal fish. Regardless of the early life treatment, PSi values were consistently high (median PSi ± interquartile range: G = 0.844 ± 0.095; SD = 0.889 ± 0.057; SP = 0.852 ± 0.048), suggesting that individuals exhibited a generalist phenotype. Yet, the evidence for the latter was not significant, as PSi values observed in our focal fish (regardless of their early life treatment) did not significantly deviate from random (Mann-Whitney U test, *N*_*1*_ = 30; *N*_*2*_ = 10,000; U = 123,553; *P* = 0.953, Fig. 1(b)).

**Fig. 2:**
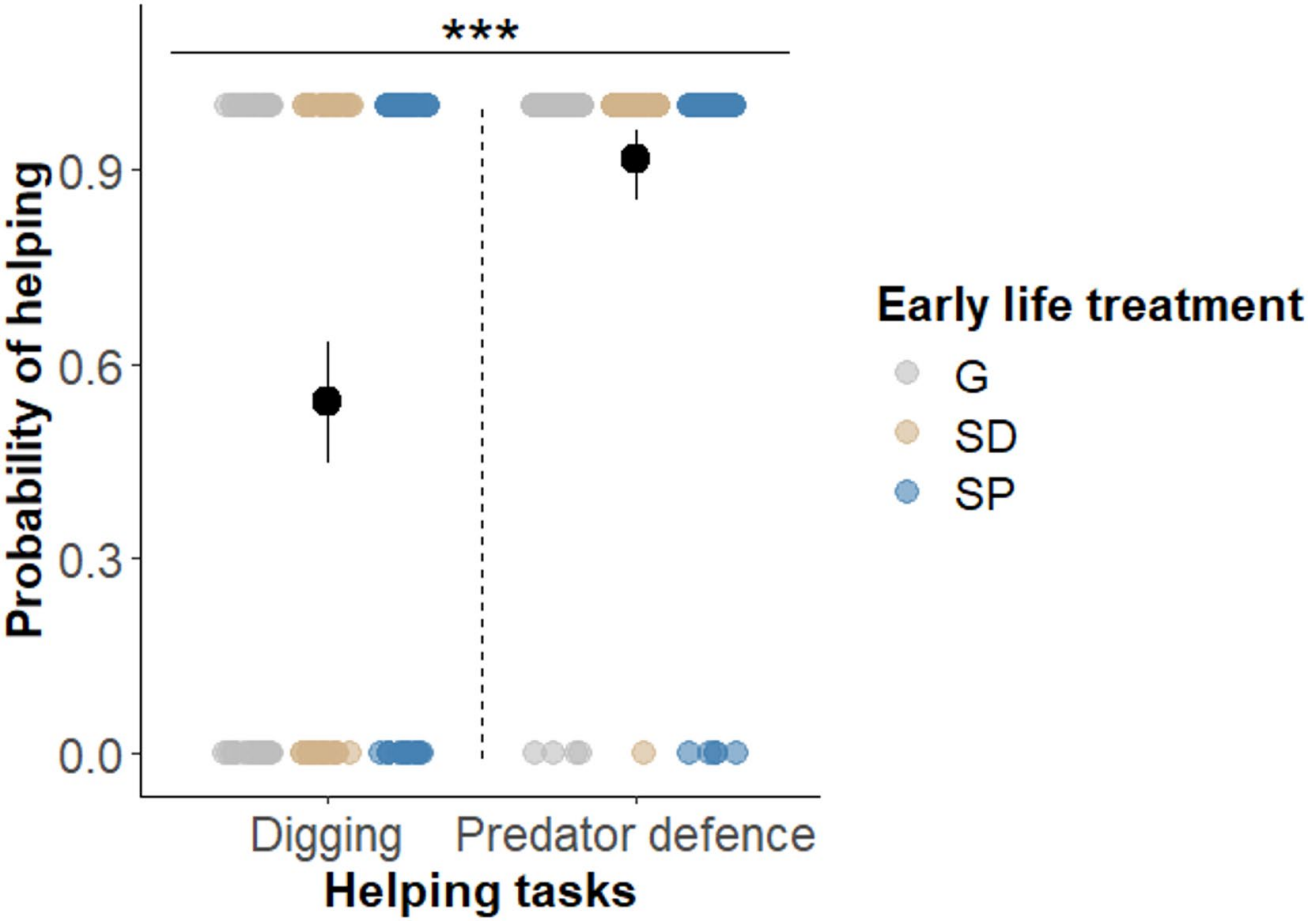
Probability of helping expressed by the focal helpers in dependence of the helping tasks and the early life treatments. The sample consisted of 10 individuals per early life treatment (G, SD, SP), with each individual completing four tests (P+/D+, P–/D–, P+/D–, P–/D+), resulting in a total of 120 data points. Mean probabilities (black dots) and Clopper-Pearson confidence intervals are shown; coloured dots show individual data points. Horizontal line and asterisks indicate significant difference (***: *P* < 0.001). Total sample sizes are given below the dots.

Effects of early-life treatment on the probability of helping were assessed by an interaction between treatments and cooperative tasks (digging vs. egg-predator defence). Consistent with the result stated above, the interaction between early-life treatment and type of help was not significant, indicating that early-life treatment did not significantly influence the probability of engaging in digging versus egg-predator defence (Type III Wald chi-squared tests: χ²_2_ = 3.245; *P* = 0.197). Additionally, there was no significant main effect of the early life treatment on the overall probability to help (χ²_2_ = 0.944; *P* = 0.624). Regardless of the early life treatment, individuals were more likely to perform egg-predator defence compared to digging (χ²_1_ = 12.452; *P* = 0.0004, Fig. 2), suggesting that individuals have preferentially engaged in defence. Helpers did not have a significantly higher probability to help when eggs were in the breeding shelter (χ²_1_ = 1.556; *P* = 0.212). The activity of egg-predator(s) did not significantly influence the probability to help (χ²_1_ = 0.005; *P* = 0.946).

## Flexibility in digging

**Fig. 3:**
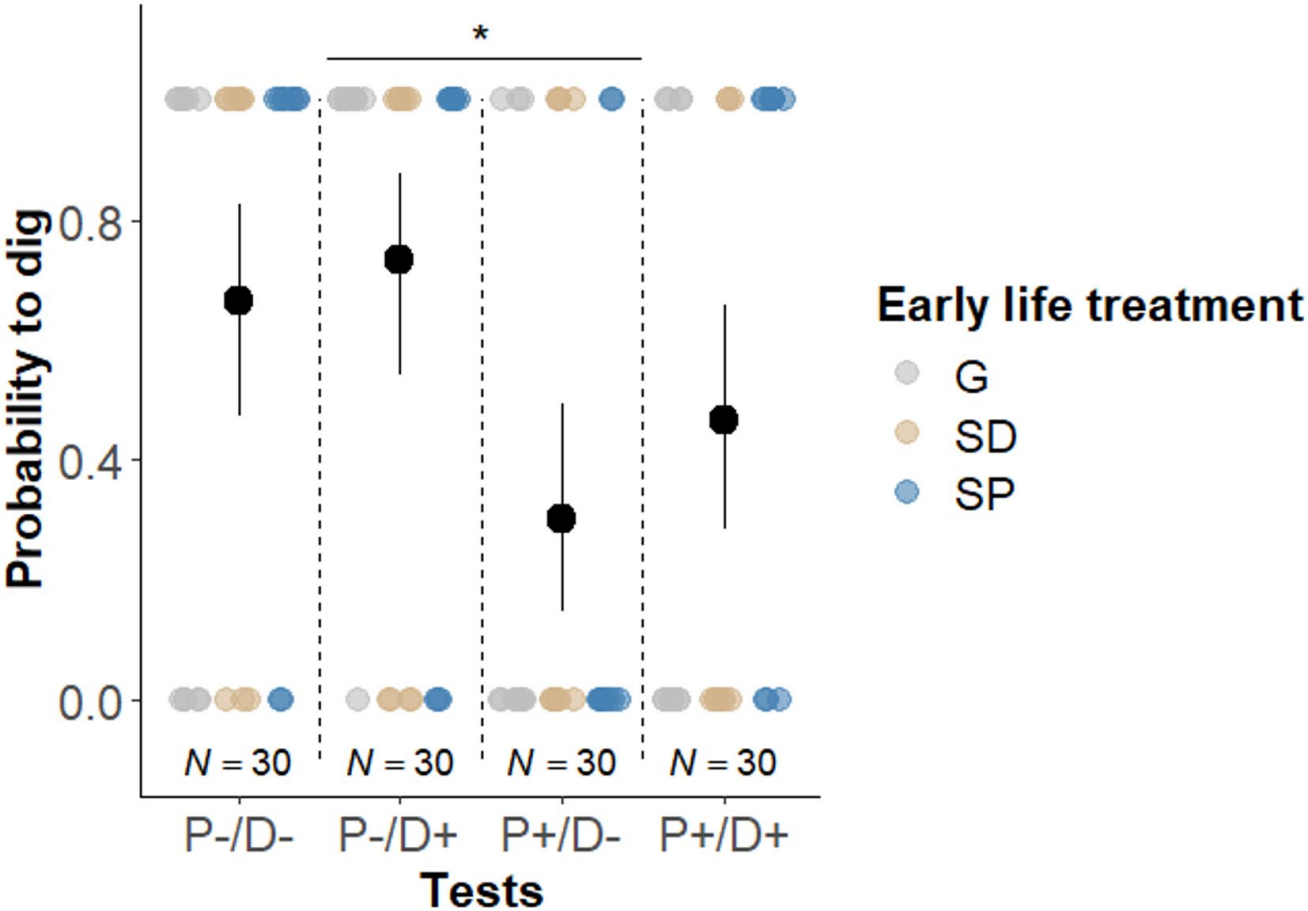
Probability of digging by the focal helper in dependence of the task allocation test and the early life treatment (G: generalist, *N =* 10; SD: specialist in digging, *N =* 10; SP: specialist in egg-predator defence, *N =* 10). Mean probabilities (black dots) and Clopper-Pearson confidence intervals are shown; coloured dots show individual data points. Horizontal line and asterisk show the significant result (*: 0.01 < *P* < 0.05). Total sample sizes are given below the dots.

**Fig. 4:**
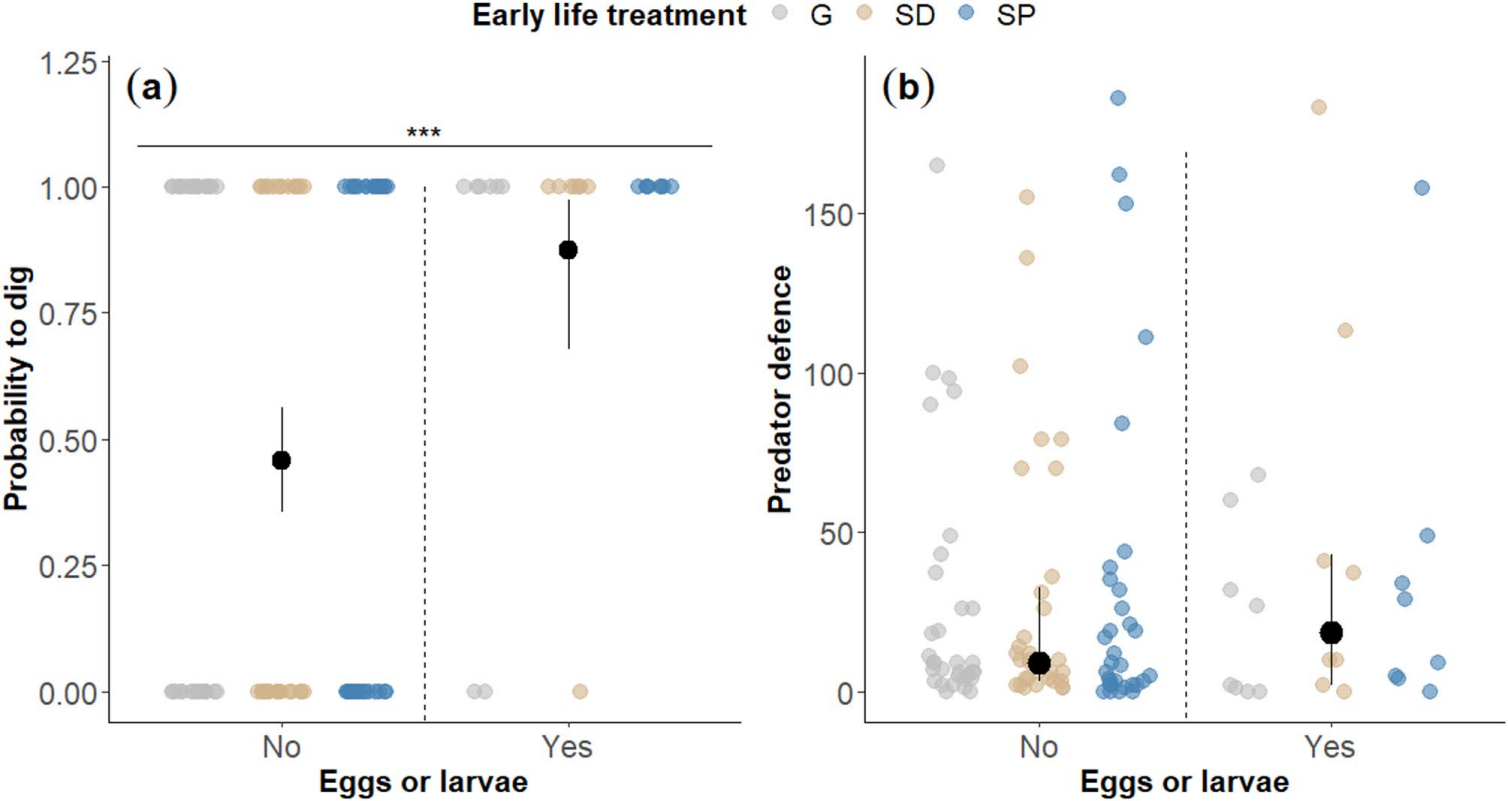
Influence of egg presence on expression of helping tasks. (a) Probability to dig of the focal helper in dependence of the egg presence and early life treatment. Mean probabilities (black dots) and Clopper-Pearson confidence intervals are shown. Horizontal line and asterisks indicate significant difference (***: *P* < 0.001). (b) Frequencies of predator defence by the focal helper in dependence of the egg presence and early life treatment. Medians (black dots) and interquartile ranges are shown; coloured dots show individual data points. The sample included 8 individuals per treatment without eggs (i.e. ‘No’) and 2 individuals per treatment with eggs (i.e. ‘Yes’), with each individual completing four tests (P+/D+, P–/D–, P+/D–, P–/D+), resulting in a total of 96 data points for the “No” condition and 24 for the “Yes” condition.

The influence of early-life treatment on the flexibility to dig was investigated through an interaction between the early life treatment and the four test situations (P+/D+; P–/D–; P+/D–; P–/D+). The probability of performing digging across the four test situations was not significantly influenced by the early life treatment (Type III Wald chi-squared tests: χ²_6_ = 8.684; *P =* 0.192). Additionally, there was no significant main effect of the early life treatment on the probability to dig (χ²_2_ = 2.520; *P* = 0.284). Nevertheless, the probability of digging significantly differed across the test situations (χ²_3_ = 10.487; *P =* 0.015, Fig. 3). Helpers had a higher probability to dig in the P–/D + situation (i.e. low need of defence and high need of digging) compared to P+/D– (i.e. high need of defence and low need of digging) (see Table 1). Therefore, our results indicate that helpers flexibly adjust their contribution to digging according to the current needs. Moreover, helpers had a higher probability of digging when they were eggs in the breeding shelter (χ²_1_ = 15.157; *P* < 0.0001, Fig. 4(a)), thereby also indicating flexibility in helping contribution. The activity of egg-predator(s) did not significantly influence the probability of digging (χ²_1_ = 1.431; *P =* 0.232).

**Table 1:** Post hoc comparisons of digging probability and amount of egg-predator defence across the four test situations, based on Tukey-adjusted z-tests. Significant results are in bold.

Parameter	Coefficient estimate	Standard error	z	*P*
**Probability to dig ~**				
P–/D– vs. P–/D+	−0.293	0.363	−0.809	0.850
P–/D– vs. P+/D–	1.089	0.442	2.460	0.066
P–/D– vs. P+/D+	0.508	0.389	1.304	0.560
P–/D + vs. P+/D–	1.382	0.452	3.058	**0.012**
P–/D + vs. P+/D+	0.801	0.401	2.000	0.188
P+/D– vs. P+/D+	−0.581	0.447	−1.299	0.564
***Amount of defence ~***				
P–/D– vs. P–/D+	0.386	0.318	1.213	0.619
P–/D– vs. P+/D–	−0.987	0.328	−3.005	**0.014**
P–/D– vs. P+/D+	−1.012	0.303	−3.340	**0.005**
P–/D + vs. P+/D–	−1.372	0.342	−4.015	**0.0003**
P–/D + vs. P+/D+	−1.398	0.313	−4.460	**< 0.0001**
P+/D– vs. P+/D+	−0.026	0.293	−0.088	0.9998

## Flexibility in egg-predator defence

**Fig. 5:**
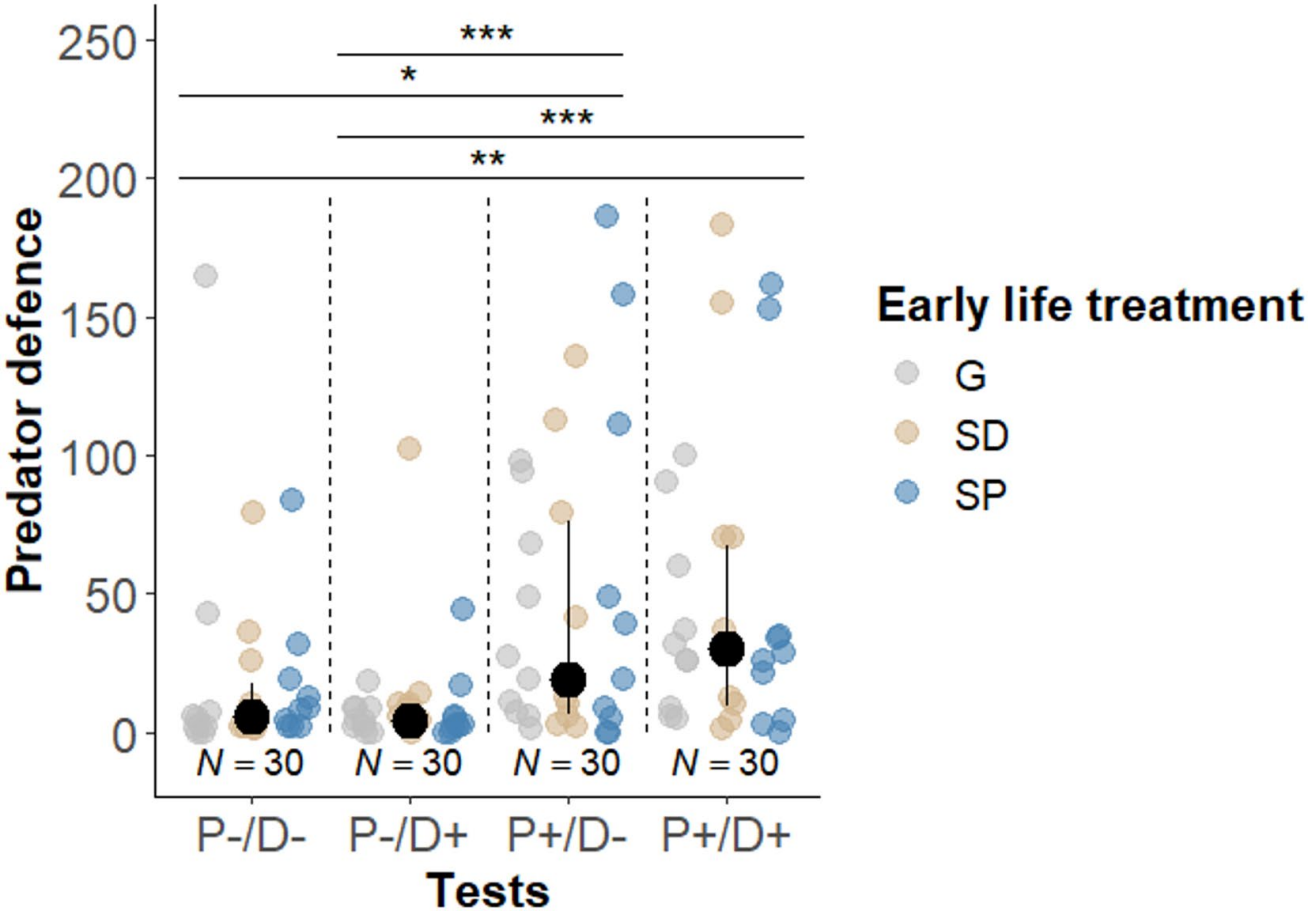
Occurrences of predator defence by the focal helper in dependence of the four test situations and the early life treatment (G: generalist, *N =* 10; SD: specialist in digging, *N =* 10; SP: specialist in egg-predator defence, *N =* 10). Medians (black dots) and interquartile ranges are shown; coloured dots show individual data points. Horizontal lines and asterisks indicate significant differences (***: 0 < *P* < 0.001; **: 0.001 < *P* < 0.01). Total sample sizes are given below the dots.

The impact of early life treatment on flexibility in defence behaviour was examined by analysing the interaction between early life treatment and the four test situations (P+/D+; P–/D–; P+/D–; P–/D+). The interaction between the early life treatment and the amount of egg-predator defence was not significant (Type III Wald chi-squared tests: χ²_6_ = 3.678; *P* = 0.720). Likewise, there were no significant main effects of early life on the contribution in predator defence (χ²_2_ = 0.296; *P* = 0.862). However, the expression of predator defence was significantly influenced by the test situation (χ²_3_ = 12.970; *P* = 0.005, Fig. 5). Helpers performed more defensive behaviours when the need to defend was high (P+, two predators close to the shelter) compared to when it was low (P–, one predator far from the shelter), regardless of the need to dig (D+, shelter filled with sand from the tank (not accessible); D–, shelter with normal sand level (accessible) (see Table 1). These results indicate that helpers flexibly adjust their contribution based on the current need for defence. Helpers did not significantly increase their predator defence when they were eggs in the breeding shelter (χ²_1_ = 0.324; *P* = 0.569, Fig. 4(b)). Also, the activity of egg-predator(s) did not significantly influence the probability of defence (χ²_1_ = 0.662; *P* = 0.416).

## Discussion

We demonstrated that early experiences of helping tasks affected neither task specialisation nor flexibility in helping contributions of *N. pulcher* helpers. Helpers were able to express both helping tasks, but they all tended to engage more in defence, albeit with no significant evidence for being specialists or generalists. Instead, individuals adjusted their defence and digging behaviours according to the current need of help induced by the helping tasks, and to increase digging behaviours when eggs were present in the breeding shelter. However, given the relatively small sample size of 10 individuals per treatment, these findings should be interpreted with caution, and further studies with larger sample sizes per treatment are needed to confirm these patterns.

Our results do not support our hypothesis that early life experiences influence future task allocation. The early experiences of a high demand for digging, or high demand for predator defence did not influence the propensity of individuals to perform a particular task or to become specialists or generalists. It also did not affect the flexibility in helping contributions. This is in line with previous findings that being raised with egg predators does not influence the expression of helping behaviour^58^. It is rather surprising to observe such a negligible effect of early experiences of cooperative needs, which is in contrast to the well-established significance of early social experiences on behavioural development in several social species^45,46^. In particular, in our study species, early social group composition and the presence of predators of adults had considerable effects on the development of behavioural phenotypes^47,52,57^.

Developmental plasticity refers to the capacity of individuals to adjust their phenotype in response to environmental conditions encountered during early life^60,61^. Therefore, early life experiences should be crucial for the development of task specialisation, as they facilitate the adaptive acquisition of information, and result in the most beneficial phenotype^40^. Indeed, early life environment influences working contribution in eusocial insects^43,44^, as well as caste differentiation in cooperatively-breeding paper wasps, sweat bees and social aphids^48–50^. Nevertheless, there is no evidence of early life effects on task specialisation in meerkats^62^ and in *N. pulcher* (this study). Therefore, these suggest that the development of specific helping phenotypes during early life is not as important in cooperatively breeding vertebrates as in cooperatively breeding invertebrates and eusocial insects. Size differences between dominants and subordinates have been observed in cooperatively breeding vertebrates^63,64^, possibly explaining variations in helping contribution^17,38,39^. Nevertheless, the growth trajectory is plastic in meerkats and *N. pulcher*^63,65^, suggesting that these species may not benefit from early-life task specialisation, but rather exhibit age polyethism^6^.

The long-term effects of early life largely hinge on the reliability and stability of early environmental cues^40,61^. However, in cases where the environment undergoes significant changes between early and adult life stages, adjusting the phenotype to early life experiences may not be optimal, and therefore may be counter-selected. In *N. pulcher*, helpers may disperse to neighbouring groups upon sexual maturity^55^, where similar environmental conditions may preserve the relevance of early life experiences. Nevertheless, some helping needs, such as predator defence or digging, are less predictable due to the sporadic nature of external disturbances like predator visits or storms. Visits of piscivorous species and egg predators, in particular, require immediate defence to mitigate risks to both adults and eggs. Moreover, when helpers disperse, they often join smaller groups^66^, where their contributions to cooperative tasks increase to meet group needs^67^. This suggests that helping behaviours should be flexible and tailored to current circumstances. Indeed, we demonstrated that helpers adjust their egg-predator defence and digging behaviours to meet current helping needs, consistent with previous studies^33–35,68^, albeit without influences of early life. Additionally, we confirmed that helpers increase territory maintenance in the presence of eggs in breeding shelters^36,67^. Consequently, helpers seem to flexibly adjust their cooperative contribution to enhance the reproductive success of the dominants^56^.

Our results may have been influenced by the choice of substrate in our early life treatment. Although *N. pulcher* populations can inhabit rocky areas without sandy bottom^69^, we opted to use sand as the substrate across all treatments, which is the substrate also present in the Kasakalawe population in Zambia from which our laboratory population has been bred. Therefore, fish raised in the ‘specialists in egg-predator defence (SP)’ treatment were raised with a sandy bottom and thus had the possibility to dig, though shelters were not filled with sand as in the other two treatments. This uniformity raises the possibility that the mere presence of sand during early development may have affected digging behaviours later in life, potentially influencing both the helping phenotype and flexibility.

The high average proportional similarity index (PSi) values indicate that helpers exhibited generalist phenotypes, although PSi values did not significantly differ from random. It is important to note that the probability of engaging in defence behaviours may have biased the PSi calculations. The PSi metric compares the proportion of time each individual spends on tasks to the average proportion spent by the population (i.e. our sample size). In our study, only five out of thirty individuals frequently engaged in digging. As a result, these individuals showed a higher deviation from the group average (i.e. high degree of specialisation, Fig. 1(a)). In contrast, the majority of individuals which primarily engaged in defence exhibited higher PSi values (i.e. low degree of specialisation). This imbalance likely influenced the PSi permutations as well, since they were based on the same unequal probabilities of defence and digging, leading to artificially inflated PSi values and an underestimation of specialisation. When group size is small, generalists may provide greater benefits to the group, as fluctuating task demands and restricted worker availability limit the effectiveness of specialisation^9,13,23^. While our PSi values do not allow us to definitively distinguish between generalist and specialist phenotypes, our findings highlight the notable flexibility of helpers in adjusting their contributions to meet immediate needs, particularly when helping alone in a small group. Therefore, to investigate effects of the early life on task specialisation, future studies should also consider the development of division of labour in larger group sizes, as this may allow individuals to allocate effort towards tasks in which they are more efficient or intrinsically more likely to perform^70^.

Furthermore, the apparent preference for defence over digging might have been influenced by our experimental group set-up, in which we presented two tasks to one fish simultaneously, and which may have been limited in attending to both tasks. Predation is a major force of natural selection, leading to the evolution of group formation and cooperation^1,71^, including in *N. pulcher*^35,72^. Additionally, the presence of egg predators increases the acceptance rate of new *N. pulcher* helpers, highlighting the benefits helpers provide in mitigating potential future threats^73^. Similarly, helpers increase defence efforts in small groups^67^. Therefore, it is likely that helpers prioritised defending against egg predators over digging, which may have been perceived as the ‘less-urgent’ task in the face of acute predation risk. This suggests that our experimental setup may have buffered the effects of the early life treatment.

We confirmed that *Neolamprologus pulcher* helpers can flexibly and adaptively adjust their helping behaviours to prevailing conditions, as previously demonstrated both in the lab and the wild^35,68^, which likely benefits the group. This flexibility may also benefit the helpers, by avoiding or reducing punishments by the dominants, when helpers have to pay in order to stay in the safety of the territory^74,75^. Flexibility in decision-making and task choice has also been highlighted in cooperatively breeding mammals, particularly through variations in vigilance behaviours in response to predation risk and the presence of pups^37,76^. Moreover, in many eusocial insects, workers flexibly adjust their roles by switching castes^10,31^. Reallocating efforts in response to environmental changes and varying helping needs offers significant benefits to social groups^77^. This is why most social species, even those with caste specialisation, retain a certain degree of flexibility^18,26,27,29^.

Cooperatively breeding cichlids represent a notable example of social complexity within the animal kingdom. Understanding the dynamics of task allocation, whether influenced by early life experiences or current needs, is pivotal for unravelling the mechanisms driving the division of labour, its evolutionary trajectory, and its implications for fitness. Our study on the cooperatively breeding cichlid *N. pulcher* highlights the significance of current needs in shaping task allocation, underscoring the importance of considering real-time challenges over early-life experiences. However, gaps remain in our understanding, particularly regarding how multiple helpers coordinate within groups. Future investigations should examine whether variation in task performance originates from individual-level traits, or whether it emerges from group-level processes. Such endeavours promise to unveil deeper insights into the underlying mechanisms of cooperative behaviours.

## Methods

### Study species

*N. pulcher* is a cooperatively breeding cichlid endemic to Lake Tanganyika in East Africa. *N. pulcher* has been widely used as a model species to examine hypotheses about the functional importance of helping, as most helpers stay in their natal territory, and if they disperse, this often happens long after reaching sexual maturity^55,66,72,74^. Cooperation in *N. pulcher* relies mostly on direct benefits helpers achieve by being allowed to stay in a group and thereby having access to the safety of a territory^72,74^. However, cooperative behaviours are enforced by coercion and punishment by dominant breeders^33,34,75^. Such punishments may be prevented through pre-emptive appeasement by increasing helping and submissive behaviours^33,68^.

A second cichlid fish species endemic from Lake Tanganyika, the egg and larvae predator *Telmatochromis vittatus*^33,34^ was used as stimulus fish for the egg-predator defence task during our early life treatments (see below), as well as during the behavioural tests later in life. These fish live solitarily, and in nature regularly visit the territories of *N. pulcher* and other substrate brooders to prey on their eggs and larvae^33,38^. Both species were maintained under housing conditions typical of those from Lake Tanganyika during the past years and the experiments (see ‘Housing conditions’).

### Housing conditions

Family groups during the early life treatment were housed in 100-L compartments of 200 L tanks equipped with ≥ 2 cm of river sand, a biological filter, four shelters at the bottom (half clay flowerpots), two floating shelters (opaque PET bottles) and one or two bundles of floating, loose plastic mesh with spaces for the fish to hide in. Following Fischer et al.^52^, the biological filter was placed in the front of each tank to decrease the odds of the dominant pair using it as a breeding shelter. During the ‘neutral phase’ (see below), sibling groups were housed in 50-L compartments of 200-L tanks, all equipped with 2 cm of sand, a biological filter and three to five clay flower pot halves as shelters. During the behavioural tests (see below), experimental groups were housed in test tanks of 100-L compartments of 200-L tanks equipped with ≥ 2 cm of sand, a biological filter, three flower pot shelters, two floating shelters and one floating bundle of plastic mesh.

*T. vittatus* individuals were housed in 200-L tanks containing ≥ 2 cm sand, two biological filters and 12 shelters at the bottom.

Natural conditions of Lake Tanganyika were simulated by a 13:11 L: D cycle, with 10 min of dimmed light at the onset and end of the light phase to simulate dawn and dusk. The water temperature was kept constant at 27 +/- 1 °C. All fish were fed 6 days per week: 5 days with commercial flake food and 1 day with a mixture of frozen zooplankton. Experimental free-swimming fry were fed with Tetramin^®^ ‘baby food’ during the 1st two months of their life, and then with Tetramin^®^ ‘junior food’ until the end of the neutral phase. During the behavioural tests, all fish were fed as described above. In addition to the normal alimentation, feeding with pieces of frozen black mosquito larvae was provided every other day three times per week to stimulate spawning by the dominant pair. This additional food provisioning occurred during the early life treatment, as well as during behavioural tests. Once eggs required for the early life treatment were obtained, any subsequent clutches were promptly removed during daily monitoring.

### Early life treatment

We used fish from the lab population of the Ethologische Station Hasli of the Institute of Ecology and Evolution, in Bern, Switzerland, which was originally derived from wild-caught fish from a population at Kasakalawe Bay, near Mpulungu, Zambia.

Experimental broods were produced in 15 family groups, consisting of a breeding pair (mean standard length (SL), i.e. from the tip of the snout to the end of the caudal peduncle of males +/- SEM = 6.39 cm +/- 0.1 cm; mean SL of females +/- SEM = 5.51 cm +/- 0.1 cm) and one helper (mean SL +/- SEM = 3.56 cm +/- 0.1 cm). *N. pulcher* groups in the laboratory are not always as stable as in the field. Therefore, the status of the helper (accepted, tolerated, or evicted, Supplementary Table S1) was verified every day. When helpers were exposed to an elevated level of aggression by the breeding pair (‘evicted’, Supplementary Table S1), the helper was removed from the tank, and the treatment continued without its presence in the family group. Three of the 15 helpers had to be removed from the family group during the first two months of treatment. All were part of the ‘specialists in digging’ treatment. Two dominants died from natural causes *after* the first two months of the treatment. As two months of early exposure to a social large group suffices to develop proper social behaviours later in life^47,52,57^, we assume that maintaining offspring with only one parent after an age of two months should have no discernible impact on subsequent helping behaviours.

**Fig. 6:**
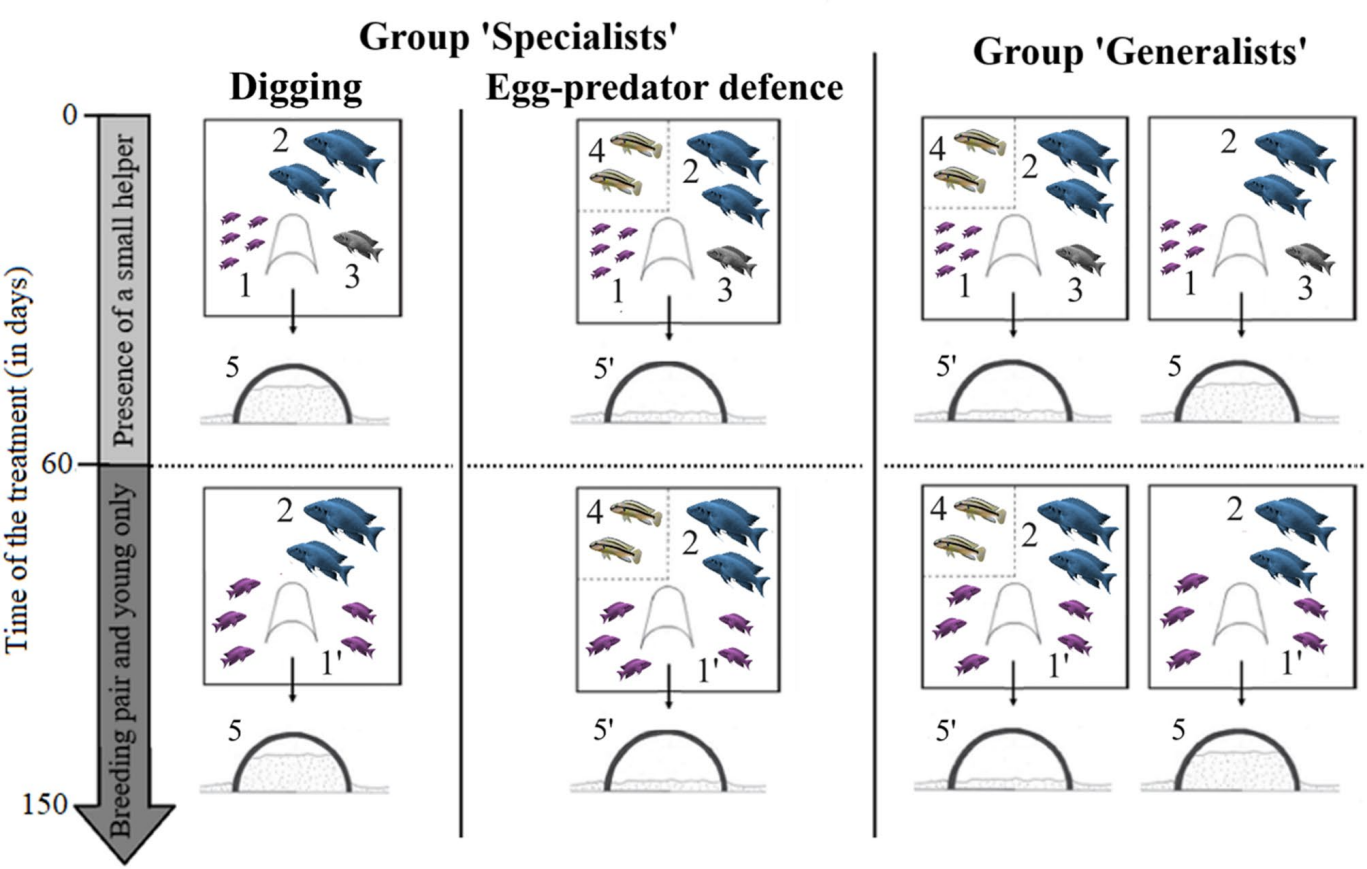
Early life treatment from day 0 to day 150 (+/- 5 days). Free-swimming fry (1) were raised with their parents (breeding pair: 2) to the juvenile stage. An unrelated helper (3) was present in the group only from day 0 to day 60 (+/- 2 days). From day 60 to day 150 (+/- 2 days), we removed the helper, so the juveniles (1’) were only kept with their parents (2). *T. vittatus* individuals (4) were presented only in the specialist group in egg-predator defence and the generalist group. Shelters were filled with additional sand (5) in the treatment ‘specialists in digging’, and left free of additional sand (5′) in the treatment ‘specialists in egg-predator defence’. In the treatment ‘generalists’, shelters were either filled with additional sand or left free of additional sand, with egg predators present only in the latter case. Figure adapted from^33^.

We waited for the breeding pair to start spawning. To avoid the potential deviation of the quality of the 1 st clutch, which can express a higher variation of egg sizes compared to later clutches (B. Taborsky, pers. obs.), we waited at least until the 2nd clutch of a pair to use it as experimental brood. We started the early life treatment once the experimental brood had reached the free-swimming stage, typically at an age of 10 or 11 days (defined as ‘day 0’). The treatment took from day 0 to day 150 (+/- 5 days). There were three early-life treatments, which involved exposure to two different cooperative tasks: territory maintenance (abbreviated ‘digging’) and defence against the cichlid *Telmatochromis vittatus*, which is a predator of eggs and larvae. The ‘digging’ task consisted of adding 75% of sand to the central shelters of a group territory and allowing the group to dig out the sand throughout the day. The ‘defence’ task entailed the introduction of two *T. vittatus* individuals near a breeding shelter for 45–60 min. The tasks were presented to the family groups 3 to 5 days per week between 0900 and 1700 (see Fig. 6), and we allowed the entire groups to engage with the task without interfering or recording individual contributions. The three early life treatments were (1) groups only experiencing the digging task; this treatment is referred to as ‘specialists in digging’ (SD, *N* = 5 family groups). (2) Groups only experiencing the egg-predator defence task ‘specialists in egg-predator defence’ (SP, *N* = 5 family groups). (3) Groups, which on each trial day encountered either the digging task or the egg-predator defence task haphazardly; that is, ‘generalists’ (G, *N* = 5 family groups). *T. vittatus* individuals were randomly taken from our lab stock population every morning and then presented individually in a transparent and perforated cylinder (radius = 5.25 cm; height = 15.5 cm) to allow for exchange of visual and chemical cues. Each *T. vittatus* individual was used in two trials per day. We used 14 individuals in total, of which each was employed both for the SP and for the G treatments. We ensured that no family was exposed to the same *T. vittatus* individuals two times in a row.

After 60 days, we removed the helper individual from the group, as the offspring reached a size where in nature they can start to contribute to helping tasks^78^. No behavioural observations were conducted during the early-life treatment phase, so we cannot confirm whether the offspring actively engaged in helping behaviours during this period. Nevertheless, free-swimming fry of *N. pulcher* start to dig and to perform aggressive behaviours within 5 weeks of age^79^; see also Camargo dos Santos & Taborsky, MS. After 150 days (± 5 days), the siblings from each of the 15 families (5 families per treatment) were measured for length. They were then housed for one month only with their siblings (i.e., ‘neutral phase’). Subsequently, individuals were marked by family group origin with one coloured elastomer implant. The marking was conducted under anaesthesia using KoiMed Sleep (Schönbach Pharmacy, Germany). Two juveniles per family group were selected to take part in the behavioural tests (see below; mean SL +/- SEM of fish of the three treatments; G: 2.71 cm +/- 0.1 cm; SD: 2.83 cm +/- 0.1 cm; SP: 2.7 cm +/- 0.1 cm). We ensured not to select the largest individual (i.e. the highest rank fish) or the smallest individual (lowest rank) from a sibling group for the behavioural tests in order to prevent strong effects of the rank on behaviours^80^.

### Tests for task specialisation and flexibility

At the age of 180 days (+/- 6 days), the 30 focal helpers took part in four behavioural tests to assess their task performance.

 We started by creating small groups of three individuals, composed of one individual reared in one of our three treatments (i.e. ‘focal helper’), and one unrelated and unfamiliar breeding pair (mean SL +/- SEM: males: 6.07 cm +/- 0.1 cm; females: 4.91 +/- 0.1 cm). For data collected during the integration of the focal helpers see the Supplementary Material (Table S1, Table S2, Table S3, Figure S1). We waited one week after the creation of groups to ensure that the focal helper was accepted by the breeding pair. The criteria for acceptance included the focal helper having complete access to the territory, and the absence of aggression from the unfamiliar breeding pair towards the helper. Otherwise, the pair was replaced by another pair, and the procedure was repeated until the focal helper was accepted. In total, we needed 50 pairs and replacement pairs to achieve the 30 stable groups (pair and focal helper). Subsequently, focal helpers were habituated to the presence of a transparent cylinder (radius = 5.25 cm; height = 15.5 cm, with perforations on the lid and on one side) by placing one empty cylinder in the test tank over five nights.

**Fig. 7:**
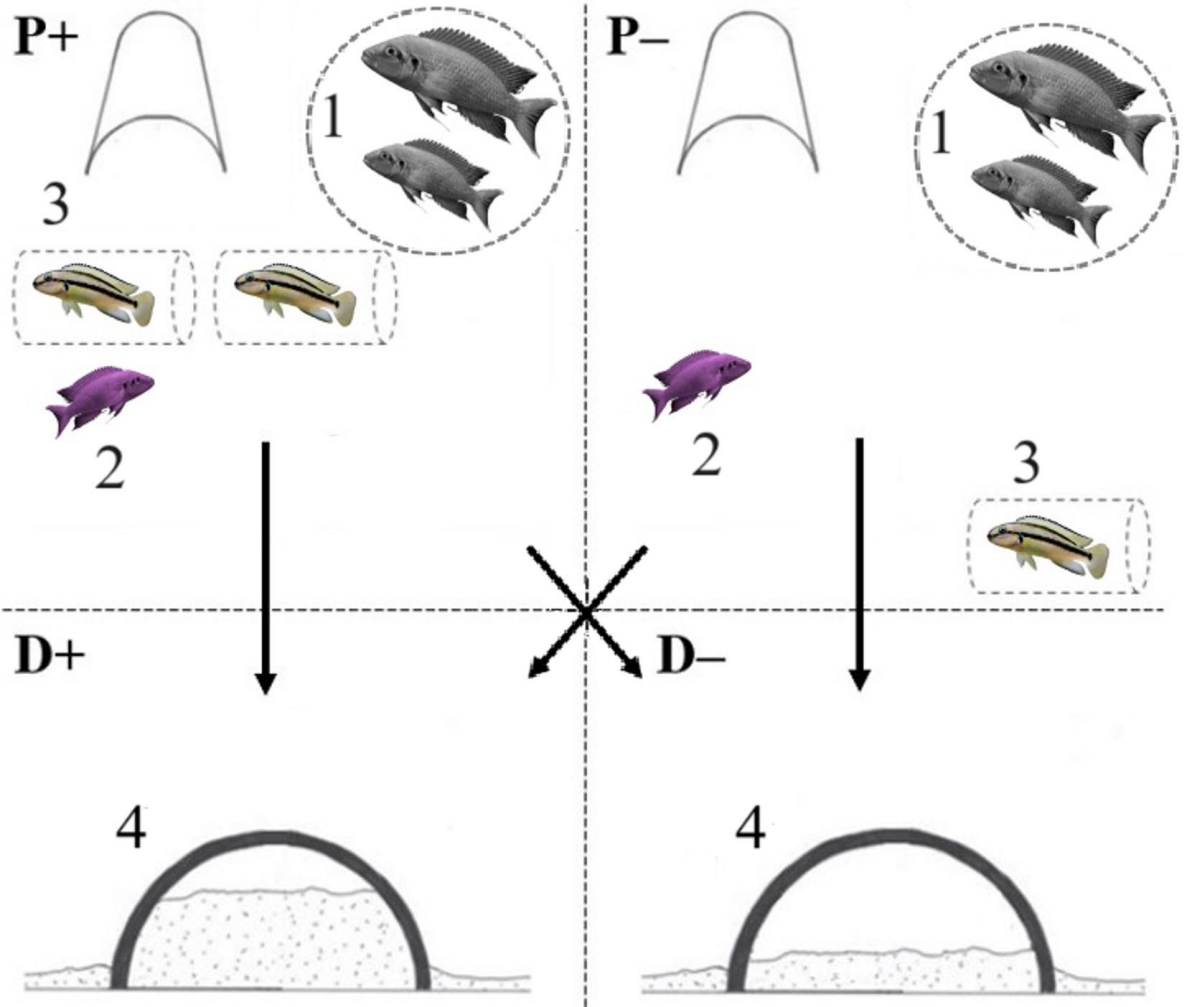
Flexibility in task allocation tests. Four possible situations are illustrated. P+: high intensity of predation; P–: low intensity of predation; D+: high intensity of digging; D–: low intensity of digging. Refer to ‘Methods’ section for explanations. (1) confined breeding pair; (2) focal helper; (3) *T. vittatus* individual; (4) test shelter. Figure adapted from^33^.

To assess whether early life influenced the helping phenotype (i.e. specialists or generalists), and the ability to adjust helping behaviours according to context (i.e., ‘flexibility’), we experimentally generated two different intensities (high = +; low = –) for digging (D) and egg-predator defence (P) to simulate varying levels of task demand in a group. Both tasks were presented simultaneously to the focal fish, thereby constituting four different combinations of intensity (‘test situations’): P+/D+, P+/D–, P–/D + and P–/D– (see Fig. 7). We hypothesised that if helpers were specialists, they would contribute preferentially to the task they specialise in (P or D) and, as a result, demonstrate flexibility only by cooperating more in the high-intensity version (+) of their specialised task compared to its low-intensity version (–). In contrast, if helpers were generalists, we expected them to contribute to both tasks and to adjust their behaviour based on intensity—cooperating more in the high-intensity trials (+) than in the low-intensity trials (–) of each task (e.g., defending more in P+/D- than in P–/D+). Furthermore, when both tasks were simultaneously high in demand (P+/D+), generalists were expected to increase their overall level of cooperation compared to when both tasks were low in demand (P–/D–). These predictions align with the idea that early-life exposure to cooperative tasks may influence the development of either a specialist or generalist helping phenotype.

High predation intensity (P+) was characterised by the presentation of two *T. vittatus* individuals put singly in two transparent and perforated cylinders (see habituation above) around 5 cm away from the breeding shelters. Low predation intensity (P–) was characterised by the presentation of one *T. vittatus* individual in a transparent and perforated cylinder around 25 cm away from the breeding shelters. High digging intensity (D+) was characterised by the addition of around 75–100% of sand in the breeding shelters. Low digging intensity (D–) was characterised by around 10% of sand put in the breeding shelters. Tests were conducted in the morning between 0900 and 1200 since *N. pulcher* individuals tend to be more active during this period (O.F., personal observation). Each focal helper faced one time each possible situation, that is four tests per fish, with a one-day resting period between each test. The order of the situations was predetermined for each fish, ensuring the sequence varied across individuals as much as possible. Each predetermined sequence was then assigned haphazardly to individuals, and the assignment was conducted blindly to treatment.

During each test, we first replaced the biological filter of the test tank with an air stone to optimise the visual conditions for video recording. To mitigate the influence of intra-group dynamics on the helper’s behaviour^33,34^, the male and female breeders were temporarily confined together in one large transparent tube placed upright and containing one of the three shelters of the test tanks (radius = 7.25 cm; height = 24.5 cm). If excessive aggression between the breeders occurred during the tests (at least 30% of the 20-minute observation period of a trial), they were individually relocated to smaller tubes (radius = 5.25 cm; height = 15.5 cm; *N =* 8). To standardise the test situations and reduce the impact of shelter selection bias, we haphazardly selected, for each test, one of the three shelters within the test tank to be allocated to the breeders in the cylinder, one to be removed entirely, and one to serve as the designated test shelter for the task. The designated test shelter was placed in a front corner of the tank (haphazardly on the right or left). If eggs were observed in one of the three shelters, this shelter was automatically designated as the test shelter for all subsequent tests. Subsequently, the test was initiated by adding sand to the test shelter in accordance with the test intensity level; and introducing one or two *T. vittatus* individuals in the cylinders, again in accordance with the intensity level, to create one of the four test situations (P+/D+, P+/D–, P–/D + and P–/D–).

Behavioural recordings were done following a 5 min period of habituation to the set-up and were done with help of video cameras (SONY^®^ Handycam^®^, Model HDR-CX405) for 20 min. Video analyses were conducted shortly after testing. We ensured that the observer (O.F.) was blind to the early life treatment of the focal fish. We measured the occurrences of each helping behaviour of the focal fish. Since predator activity is likely to influence helping behaviours^81^, we measured the frequency of predator movement by recording whether the predators were swimming (yes/no) every 30 s. This measure of activity was later included in our model to control for the motivation to defend. We tested 30 *N. pulcher* focal individuals (G: *N =* 10; SP: *N =* 10; SD: *N =* 10), and used a total of 37 *T. vittatus* individuals. Every week, *T. vittatus* individuals were randomly selected from our lab stock population and kept alone in ‘isolation nets’ suspended next to each other under the surface of an empty tank. This arrangement was maintained for the duration of the four tests, which spanned one week. Each *T. vittatus* individual was used one time per day. For each focal individual tested, we ensured that the same egg-predator was never used more than once across the four tests (i.e., 6 different egg-predators for each individual). Additionally, the combination of six egg predators used for each individual was unique. All behavioural recordings were analysed with the Behavioural Observation Research Interactive Software^82^.

### Statistics

Statistical analyses were performed with the R software, version 4.3.3^83^.

Previous studies with *N. pulcher* reported size-based task performance and division of labour, with fish ranging from 2.5 cm to 3.5 cm defending more, and fish larger than 3.5 cm digging more^38^. As all focal fish in our trials were of similar size (range 2.3 cm to 3.0 cm), we did not include size in our models to improve statistical power, given our small sample size. Six pairs laid eggs right before the test (G: *N =* 2, SD: *N =* 2; SP: *N =* 2). As the presence of eggs is known to influence task performance^36,67^, we included their presence or absence in all statistical models. To analyse the helping performance and potential task specialisation we fitted four models.

We first investigated whether the early life treatments influenced the helping phenotype of our focal fish, specifically examining if they exhibited specialist or generalist phenotypes. To do so, we calculated the degree of specialisation of each individual by using the proportional similarity index PSi^84^. The PSi is used to quantify the deviation in individuals’ behaviour relative to the population. Especially, its use has provided insights into diet specialisation in various animal taxa^85–87^. However, its application to task specialisation remains limited, with only one study using the PSi to investigate individual task specialisation in the common wasp (*Vespula vulgaris*)^88^. Initially, we determined the total proportion of time helpers spent digging (*p*_*digging*_) and defending against egg predators (*p*_*defence*_) across the four tests. We then calculated the average proportions of digging ($\:\stackrel{-}{p}$_*digging*_) and defence ($\:\stackrel{-}{p}$_*defence*_) across all individuals:

$$\:{\stackrel{-}{p}}_{digging}=\:\frac{1}{N}\sum\:_{i=1}^{N}{p}_{i,\:digging}$$

$$\:{\stackrel{-}{p}}_{defence}=\:\frac{1}{N}\sum\:_{i=1}^{N}{p}_{i,\:defence}\:$$

Next, we computed the proportional similarity index for each individual *i*:

 

$${\rm PSi}_i = \:1\:-\:0.5[\left|{p}_{i,\:digging}-\:{\stackrel{-}{p}}_{digging}\right|+\left|{p}_{i,\:defence}-\:{\stackrel{-}{p}}_{defence}\right|]$$

Therefore, PSi varies between 0 (high degree of specialisation) to 1 (low degree of specialisation). To assess whether individual specialisation was statistically significant, we performed a permutation test. Specifically, we randomised the total counts of digging and defence tasks across individuals and recalculated the task proportions and corresponding PSi values for each permutation. This process was repeated 10,000 times to create a null distribution of PSi values. We then compared the observed PSi values to the permuted distribution using a Mann-Whitney U test, implemented in the *stats* package^83^.

To test the influence of the early life treatment on the PSi of our focal individuals, we fitted a linear mixed-effects model (LMM). The model included the PSi as the response variable and the early life treatment as a fixed effect. We included the pair of origin of the focal fish as a random factor. Visual inspection of residuals indicated deviations from both homoscedasticity and normality. Therefore, the P-value was obtained using a permutational multivariate analysis of variance (permanova) with the package *predictmeans*^89^.

Next, we asked which task helpers performed more frequently (digging or defence) in dependence of early life experience. Towards that aim, we fitted a generalised linear mixed-effects model (GLMM) assuming a binomial distribution to test for an effect of the early life treatment on the probability to perform a helping task (1/0, response variable), using the package *lme4*^90^. The model included the interaction between the type of helping task during the test trials (i.e. digging or defence) and the early life treatment, egg presence (yes/no), and the activity of egg-predator(s) as fixed effects. We included the identity of the focal fish and pair of origin as random factors. ‘Pair of origin’ was subsequently removed, as it did not explain any variance. Assumptions of normality and homoscedasticity were tested using simulation-based diagnostics provided by the *DHARMa* package^91^, confirming that model residuals met these assumptions. They were not overdispersed (package *performance*^92^). There was no multicollinearity detected among the variables. P-values were obtained using type III Wald chi-squared tests, implemented via the Anova() function from the R package *car*^93^.

We examined how the early life treatment affected the helpers’ ability to flexibly adjust their digging behaviours, as indicated by changes in their digging performance across the four test situations (P+/D+; P+/D–; P–/D+; P–/D–). This occurrence of flexibility was examined by fitting a GLMM assuming a binomial distribution, using the package *lme4*^90^. The response variable was the probability of digging behaviours in the focal fish. As fixed effects, we included the interaction between the early life treatment and the test situation, the activity of egg-predator(s), and egg presence (yes/no). The random factors ‘identity of the focal fish’ (to account for repetitions) and ‘pair of origin of the focal fish’ were also included in the model. Residuals did not deviate from normality and homogeneity, and were not overdispersed. Also, there were no multicollinearities among the variables. P-values were obtained using type III Wald chi-squared test with the Anova() function from the R package *car*^93^. Results of the tests were investigated using least square means post hoc comparisons with Tukey correction, implemented in the R package *emmeans*^94^.

Correspondingly, we also investigated whether the early life treatment influenced the ability to adjust defence behaviours across our four test situations. Flexibility to defend against the egg predator was examined by using a GLMM assuming a Poisson distribution for count data, using the package *lme4*^90^. As the residuals of the initial model were overdispersed, we assumed a negative binomial distribution. The response variable was the number of predator defence expressed by the focal fish. We included the same fixed factors and random factors as in the model on digging flexibility. Residuals were normally distributed and homogeneous (package *DHARMa*^91^. There were no multicollinearities among all variables. Extraction of P-values and calculation of *post-hoc* comparisons were done as in the previous model.

### Ethical note

All experiments were conducted at the Ethological Station Hasli of the Institute of Ecology and Evolution of the University of Bern, Switzerland. The captive holding of the fish was approved under licence no. BE 4/22 of the Veterinary Office of the Canton of Bern. The experiments were also approved under licence no. BE 34/22 of the Veterinary Office of the Canton of Bern. None of the individuals was injured during the entire study. We meticulously observed family groups (during the early life treatment) and experimental groups (during the behavioural tests) at least three times daily to ensure the absence of stress indicators such as changes in colour patterns, postures, scale condition, and position of the fish in the tank. We also monitored interactions between the focal fish and the unrelated breeding pair to prevent excessive aggression from dominants, which could have confined fish to a corner of the tank and/or caused injury to their scales and fins. All fish behaved normally after the end of the test trials, and they were right after the end of the trials transferred to aggregations in 200-L tanks, together with fish of the same age and early life background. Two large dominant fish died from natural causes during the early life treatment. We followed the guidelines provided by the Association for the Study of Animal Behaviour/Animal Behaviour Society for the treatment of animals in behavioural research and Teaching^95^. All methods were also reported in accordance with the ARRIVE 2.0 guidelines^96^. All fish were retained in our permanent breeding stock after the end of the study.

## Supplementary Information

Below is the link to the electronic supplementary material.

Supplementary Material 1

Supplementary Material 2

## Data Availability

The datasets generated and/or analysed during the current study are available on the Open Science Framework (OSF) repository: 10.17605/OSF.IO/75EV9.
