# Supplementary material for: Task allocation in a cooperative breeder reflects current needs, not early-life experience
Source: Sci Rep. 2025 Oct 22;15:36851. doi: 10.1038/s41598-025-20618-1 (PMC12546903; doi:10.1038/s41598-025-20618-1)
Supplement: Supplementary file 1 — Supplementary Material 1 [file 41598_2025_20618_MOESM1_ESM.pdf]

Supplementary

Task allocation in a cooperative breeder reflects current needs, not early-life experience

Océane Vanessa Ferreira*, Barbara Taborsky

Division of Behavioural Ecology, Institute of Ecology and Evolution, University of Bern, Switzerland

*** Correspondence**

Océane Ferreira

oceane.ferreira@unibe.ch

Wohlenstrasse 50a, CH-3032 Hinterkappelen, Switzerland

Fr +33: 0679180683

E-mail addresses and ORCID numbers

OF: oceane.ferreira@unibe.ch; ORCID: 0009-0005-8504-9501

BT: barbara.taborsky@unibe.ch; ORCID: 0000-0003-1690-8155

Integration test

Method

Behavioural tests

When juveniles were 180 +/- 6 days old, we tested their ability to be accepted by an unrelated breeding pair, and to become a brood care helper (i.e. 'integration test')[1]. We followed the method described in previous papers[2,3].

Dominant pairs were always at least 1 cm larger than the focal fish (mean SL +/- SEM: males: 6.07 cm +/- 0.1 cm; females: 4.91 +/- 0.1 cm). Each focal fish (sample size: generalist: N = 10; specialist in egg-predation defence: N = 10; specialist in digging: N = 10) spent a night singly in a 100-L tank equipped with sand, a biological filter, three shelters at the bottom, and two floating shelters (to allow the focal fish to hide and escape aggressive behaviours). In the following morning, we first replaced the biological filter by an air stone (to increase the observation capacity of the test tank). Then, we added the unrelated and unfamiliar breeding pair in the test tank, and we started to video-record all behavioural activities for 30 minutes, using a camera (SONY® Handycam®, Model HDR-CX405). We ensured that the observer (O.F.) was blind to the early life treatment of the focal fish. We measured: (1) aggressive and submissive behaviours between the focal fish and the members of the breeding pair (see Table S1 for the behavioural repertoire that was scored); (2) the status of the focal individual 4 hours and 24 hours after the integration test (Table S2).

When the focal fish was accepted as a brood care helper after 24 hours, the group was maintained to conduct the behavioural tests on task allocation. Otherwise, the pair was changed until the helper was accepted. In total, 50 pairs have been used for the experiment.

Statistical analysis

Statistical analyses were performed with the R software, version 4.3.3[4].

We tested the ability of the focal helpers to be accepted as a brood care helper by an unrelated breeding pair by analysing two different outcome variables: (1) the total number of submissions expressed; (2) the probability of acceptance at the end of the test. We examined the effect of the early life treatment on the number of submissions expressed (response variable) with three linear mixed models, using the package *lme4*[5]. Each model included the interaction between the early life treatment and one of three aggression measures -total

aggression received, overt aggression received, or restrained aggression received- as fixed effects, along with the size of the focal fish. The pair of origin of the focal fish was also included in the model as a random factor. Assumptions of normality and homoscedasticity were assessed by visual inspection of Q–Q plots and Tukey–Anscombe plots (residuals vs. fitted values). No multicollinearity was detected among predictors. We therefore applied Type III Wald chi-square tests using the Anova() function from the *car* package[6]. The interaction term was not significant in any of the models (total aggression × treatment: $\chi^2_2 = 0.785$, $P = 0.675$; overt aggression × treatment: $\chi^2_2 = 0.752$, $P = 0.687$; restrained aggression × treatment: $\chi^2_2 = 0.164$, $P = 0.922$), and was subsequently removed. Final models were tested using Type II Wald chi-square tests. We calculated the partial R^2 of each significant predictor by comparing the full model to a reduced model excluding the predictor of interest. Marginal R^2 values were computed using the r.squaredGLMM() function from the *MuMIn* package[7]. The difference in marginal R^2 between the full and reduced models was interpreted as the proportion of variance in submission uniquely explained by the predictor.

Next, we examined the effects of the early life treatment on the status of the focal helper (i.e., 'accepted', 'tolerated' or 'evicted') after 4- and 24-hours using Fisher exact tests from the package *stats*[4].

Results

The submission expressed by focal helpers was not significantly influenced by the interaction between early life treatment and the aggression they received, regardless of whether total, overt, or restrained aggression was measured (Table S3). Similarly, early life treatment alone did not significantly affect the level of submission in any of the three models.

While there was no significant correlation between submission and either total or overt aggression received, a significant positive association was found between submission and restrained aggression (Figure S1). Restrained aggression explained approximately 9.8% of the variance in submission expressed (marginal $R^2 = 0.098$).

Helper body size had no effect in the model including restrained aggression, but in the models accounting for total aggression and overt aggression, larger helpers submitted significantly more (Table S3). Size explained approximately 9.5% of the variance in

submission expressed in the model with total aggression (marginal R^2 = 0.095), and approximately 13.9% in the model with overt aggression (marginal R^2 = 0.139).

Seventeen helpers were accepted four hours after the integration test (generalist group: N = 4; specialist in digging group: N = 6; specialist in egg-predation defence: N = 7), four were tolerated (generalist group: N = 1; specialist in digging group: N = 2; specialist in egg-predation defence group: N = 1), and nine were evicted (generalist group: N = 5; specialist in digging group: N = 2; specialist in egg-predation defence group: N = 2). A day after the integration, sixteen helpers were accepted by a breeding pair (generalist group: N = 5; specialist in digging group: N = 5; specialist in egg-predation defence group: N = 6) , five were tolerated (generalist group: N = 1; specialist in digging group: N = 2; specialist in egg-predation defence group: N = 2), and nine were still evicted (generalist group: N = 4; specialist in digging group: N = 3; specialist in egg-predation defence group: N = 2). The early life treatment did not significantly influence these status four hours after the integration (Fisher exact test: P = 0.624), nor twenty-four hours after the integration (Fisher exact test: P = 0.945).

Table S1: Behavioural repertoire used during the integration test

Category	Behaviour	Description
**Overt aggression (attacks)**	Ramming; bow swimming	Very fast linear approach towards another fish ending with physical contact (ramming); may be a dash to and fro, with hitting the other fish at the apex of a bow-shaped swimming trajectory (bow swimming)
	Biting, Chasing	Biting another fish or attempting to do so (with physical contact)
**Restrained aggression (displays)**	Fin spread + head down display	All fins, particularly the unpaired fins, are maximally spread, body kept in a stiff posture; body may be tilted with head pointing downwards (head down display)
	Frontal approach; operculum spread	Linear approach towards another fish that is abruptly stopped before physical contact; usually in combination with spreading of opercula and lowering the branchiostegal membrane. Both components, frontal approach and operculum spread may also occur without one another
**Submissive**	Tail quiver	Caudal peduncle, tail fin and back end of dorsal fin are intensively vibrating while the unpaired fins are folded; body may be pressed on the ground
	Zig-zag swimming	Swimming in short bursts in a zig-zag pattern in front of a (usually dominant) fish

Table S2: Criteria to determine three helper statuses used during the early life treatment and the integration test

	HELPER STATUS		
	**Accepted**	**Tolerated**	**Evicted**
**Behavioural characteristics**	-Total access to the tank<br>-No / few attacks even when close to the shelters	-Access to a limited area of the tank<br>-Attacks when close to the dominants (< 3 cm)	-Confined to a corner of the tank<br>-Heavily attacked by the dominants

Table S3: Results of the models analysing the total number of submissions expressed in the integration test, based on type II Wald chi-squared tests for the three models. Significant results are in bold.

Fixed variable	χ^2	***Df***	***P***
~ Total aggression			
Treatment	0.107	2	0.948
Total aggression	0.077	1	0.782
Size	4.314	1	**0.038**
~ Overt aggression			
Treatment	0.087	2	0.957
Overt aggression	1.332	1	0.249
Size	6.649	1	**0.0099**
~ Restrained aggression			
Treatment	0.212	2	0.899
Restrained aggression	5.113	1	**0.024**
Size	1.948	1	0.163

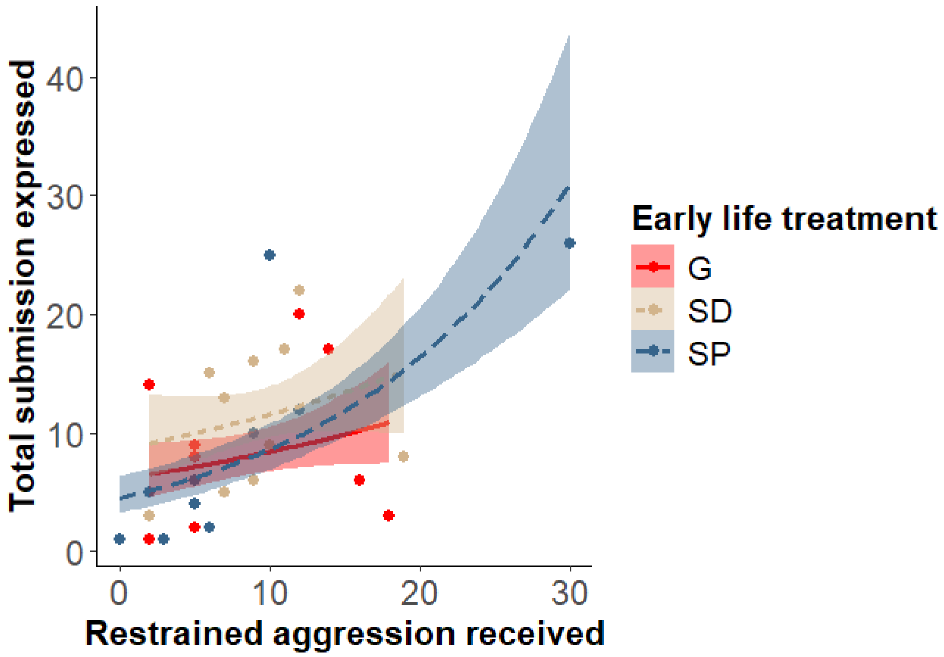

Figure S1: Submission expressed by the focal helper in dependence of the amount of restrained aggression it has received during the integration test, and the three early life treatments (G: generalist, N = 10; SD: specialist in digging, N = 10; SP: specialist in egg-predation defence, N = 10). Coloured dots represent individuals from the different treatments. Regression lines and confidence intervals are shown for each treatment, but they were not significantly different from each other.

REFERENCES

1. Taborsky, M. & Limberger, D. Helpers in fish. *Behav. Ecol. Sociobiol.* **8**, 143–145. https://doi.org/10.1007/BF00300826 (1981).

2. Ferreira, O. V., Szabo, B. & Taborsky, B. Effects of the early social environment on behavioural flexibility in a cooperatively breeding cichlid fish. *Anim. Behav.* **219**. https://doi.org/10.1016/j.anbehav.2024.11.015 (2025).

3. Taborsky, B., Arnold, C., Junker, J. & Tschopp, A. The early social environment affects social competence in a cooperative breeder. *Anim. Behav.* **83**, 1067–1074. https://doi.org/10.1016/j.anbehav.2012.01.037 (2012).

4. R Core Team. R: A Language and Environment for Statistical Computing. *R Foundation for Statistical Computing, Vienna, Austria.* https://www.R-project.org/ (2024).

5. Bates, D., Maechler, M., Bolker, B. & Walker, S. Fitting Linear Mixed-Effects Models Using lme4. *J. Stat. Softw.* **67**, 1–48. https://doi.org/10.18637/jss.v067.i01 (2015).

6. Fox, J. & Weisberg, S. *An R companion to applied regression*. https://CRAN.R-project.org/package=car (2019).

7. Bartoń, K. MuMIn: MultiModel Inference. R package version 1475. https://CRAN.R-project.org/package=MuMIn (2023).
