# Supplementary figures and images for: Task allocation in a cooperative breeder reflects current needs, not early-life experience
Source: Sci Rep. 2025 Oct 22;15:36851. doi: 10.1038/s41598-025-20618-1 (PMC12546903; doi:10.1038/s41598-025-20618-1)

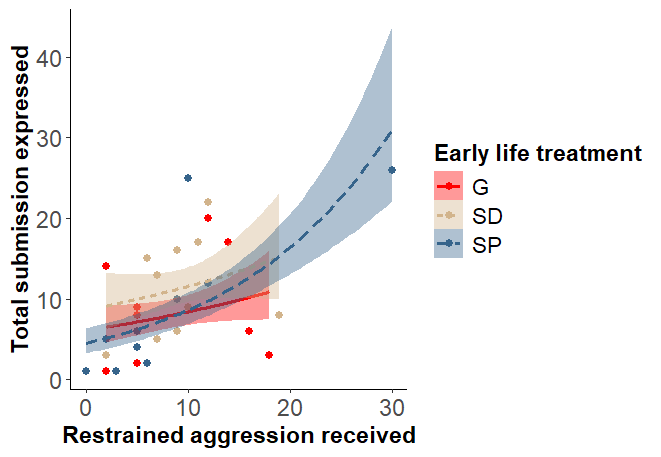

Supplement: Supplementary file 2 — Supplementary Material 2 [file 41598_2025_20618_MOESM2_ESM.tiff]
